# In vivo modeling recapitulates radiotherapy delivery and late-effect profile for childhood medulloblastoma

**DOI:** 10.1093/noajnl/vdae091

**Published:** 2024-06-06

**Authors:** Jemma Castle, Gary Shaw, Dominic Weller, Edward Fielder, Teklu Egnuni, Mankaran Singh, Roderick Skinner, Thomas von Zglinicki, Steven C Clifford, Susan C Short, Satomi Miwa, Debbie Hicks

**Affiliations:** Wolfson Childhood Cancer Research Centre, Newcastle University Centre for Cancer, Translational and Clinical Research Institute, Newcastle University, Newcastle upon Tyne, UK; Leeds Institute of Medical Research, Wellcome Trust Brenner Building, St James’s University Hospital, Beckett St, Leeds, UK; Wolfson Childhood Cancer Research Centre, Newcastle University Centre for Cancer, Translational and Clinical Research Institute, Newcastle University, Newcastle upon Tyne, UK; Biosciences Institute, Campus for Ageing and Vitality, Newcastle University, Newcastle upon Tyne, UK; Leeds Institute of Medical Research, Wellcome Trust Brenner Building, St James’s University Hospital, Beckett St, Leeds, UK; Wolfson Childhood Cancer Research Centre, Newcastle University Centre for Cancer, Translational and Clinical Research Institute, Newcastle University, Newcastle upon Tyne, UK; Wolfson Childhood Cancer Research Centre, Newcastle University Centre for Cancer, Translational and Clinical Research Institute, Newcastle University, Newcastle upon Tyne, UK; Biosciences Institute, Campus for Ageing and Vitality, Newcastle University, Newcastle upon Tyne, UK; Wolfson Childhood Cancer Research Centre, Newcastle University Centre for Cancer, Translational and Clinical Research Institute, Newcastle University, Newcastle upon Tyne, UK; Leeds Institute of Medical Research, Wellcome Trust Brenner Building, St James’s University Hospital, Beckett St, Leeds, UK; Biosciences Institute, Campus for Ageing and Vitality, Newcastle University, Newcastle upon Tyne, UK; Wolfson Childhood Cancer Research Centre, Newcastle University Centre for Cancer, Translational and Clinical Research Institute, Newcastle University, Newcastle upon Tyne, UK

**Keywords:** late-effects, medulloblastoma, modelling, radiotherapy, survivorship

## Abstract

**Background:**

Medulloblastoma (MB) is the most common malignant pediatric brain tumor, with 5-year survival rates > 70%. Cranial radiotherapy (CRT) to the whole brain, with posterior fossa boost (PFB), underpins treatment for non-infants; however, radiotherapeutic insult to the normal brain has deleterious consequences to neurocognitive and physical functioning, and causes accelerated aging/frailty. Approaches to ameliorate radiotherapy-induced late-effects are lacking and a paucity of appropriate model systems hinders their development.

**Methods:**

We have developed a clinically relevant in vivo model system that recapitulates the radiotherapy dose, targeting, and developmental stage of childhood medulloblastoma. Consistent with human regimens, age-equivalent (postnatal days 35–37) male C57Bl/6J mice received computerized tomography image-guided CRT (human-equivalent 37.5 Gy EQD2, *n* = 12) ± PFB (human-equivalent 48.7 Gy EQD2, *n* = 12), via the small animal radiation research platform and were longitudinally assessed for > 12 months.

**Results:**

CRT was well tolerated, independent of PFB receipt. Compared to a sham-irradiated group (*n* = 12), irradiated mice were significantly frailer following irradiation (frailty index; *P* = .0002) and had reduced physical functioning; time to fall from a rotating rod (rotarod; *P* = .026) and grip strength (*P* = .006) were significantly lower. Neurocognitive deficits were consistent with childhood MB survivors; irradiated mice displayed significantly worse working memory (Y-maze; *P* = .009) and exhibited spatial memory deficits (Barnes maze; *P* = .029). Receipt of PFB did not induce a more severe late-effect profile.

**Conclusions:**

Our in vivo model mirrored childhood MB radiotherapy and recapitulated features observed in the late-effect profile of MB survivors. Our clinically relevant model will facilitate both the elucidation of novel/target mechanisms underpinning MB late effects and the development of novel interventions for their amelioration.

**Key Points**

1. In vivo model system recapitulates the radiotherapy dose, targeting, and developmental stage of childhood medulloblastoma.
2. Irradiated mice display MB-like deficits to neurocognitive and physical functioning, and frailty, independent of receipt of PFB dose.

**Importance of the Study**

Eighty percent of children diagnosed with a brain tumor now become 5-year survivors, driven by the delivery of combination and intensified treatments. This exposure to intensive treatments leaves pediatric brain tumor survivors at increased risk of detrimental life-long late effects associated with their disease and its therapy. Medulloblastoma (MB) survivors are particularly burdened due to the routine use of curative high-dose regimens that include irradiation to the whole brain (CRT) plus an additional posterior fossa boost (PFB) dose. The development of effective pharmacological or other interventions aimed at prevention/treatment of therapy-associated deficits is a major clinical goal; however, a paucity of appropriate model systems hinders their development. Our highly disease-relevant model recapitulates childhood MB radiotherapy dose, targeting, and late-effect profile, at an equivalent developmental stage. Thus, our clinically relevant model provides an essential platform that will both facilitate the elucidation of novel/target mechanisms underpinning MB late effects and the development of novel neuro-interventional strategies.

Intensified therapies for children with cancer have led to 5-year survival rates approaching 85%^1^; however, this has come at a huge cost. Survivors have a high risk of developing life-changing or life-threatening late effects as a result of cancer treatment that affects the majority of physiological and psychosocial systems; examples include cardiotoxicity, renal toxicity, ototoxicity, endocrine impairment, subsequent malignancies, neurocognitive deficits, impaired neuromuscular function, and accelerated aging.^2–6^

Cranial radiotherapy (CRT), the mainstay of pediatric brain tumor treatment in non-infants, is a causative factor in lasting neurocognitive deficits, with survivors suffering intellectual disability, low levels of academic attainment, poor psychosocial satisfaction, and reduced independence in adulthood.^7–9^ Many survivors experience impaired physical functioning,^10^ and neurological difficulties such as ataxia and co-ordination disorders, as well as reduced fine motor skills.^11^ While these deficits present and persist in the years following treatment, others have a longer latency; adult survivors of childhood brain tumors develop numerous co-morbidities^12,13^ and become more frail over their life course.^14^

Medulloblastoma (MB), the most common malignant pediatric brain tumor, is typically treated with high doses of craniospinal irradiation (CSI) including CRT (up to 36 Gy) with a posterior fossa boost dose (PFB; total dose up to 54 Gy).^15^ CSI is omitted from treatment protocols for the very youngest patients due to its intolerable toxicity and late-effect profile in this age group and is reserved for children aged over either 3 or 5 years, depending on national treatment philosophies. High-dose CRT promotes the greatest intellectual impairment; the mean loss of IQ points is between 2.5 and 3.9 per year,^16–18^ reaching a plateau of impairment around 2 standard deviations below average. 70%–90% of this group demonstrate a significant impairment in global intellectual functioning; many survivors experience attention deficits, slower processing speed, and impaired working memory,^11,19–21^ which are in turn strongly correlated with decreased quality of life.^22–25^

There is a critical need for the development of interventions to prevent or ameliorate the MB late effects that burden survivors throughout their adolescent and adult life.^12,13^ However, the development of such interventions is hindered by a lack of suitable experimental model systems. Previous in vivo cranial irradiation studies have typically failed to fully model pediatric brain tumor regimens (Supplementary Table 1). Many have been limited by a common tradeoff, which was either the use of clinically relevant radiotherapy doses in adult mice^26–32^ or sub-relevant doses in juvenile mice.^33–38^ Furthermore, traditional approaches did not allow for the delivery of targeted cranial irradiation, and instead typically employed whole-head irradiation, incorporating vulnerable structures such as the ears, eyes, and mouth in the radiation field,^27,33,39–42^ resulting in high levels of acute toxicity with little clinical relevance.^43,44^ Moreover, non-targeted radiation approaches are not able to deliver radiation specifically to substructures of the brain, and therefore cannot recapitulate the PFB commonly used in MB radiotherapy regimens. Previous studies have also typically focused solely on neurocognition to the exclusion of other facets of the late-effect profile, and have rarely characterized models beyond 6 months post-irradiation, therefore failing to describe the chronic burden over the life course.^27,28,41,45–47^ To address these limitations, we developed a novel in vivo model through the combination of the delivery of MB-like fractionated high-dose radiotherapy to juvenile mice, to mirror those MB treatment paradigms that result in the greatest risk of severe late-effects. We performed longitudinal assessments across the life course up to human-equivalent middle age, to model the long-term burden suffered by survivors of childhood brain tumors.

## Materials and Methods

### Mice

Juvenile male C57BL/6J mice (*n* = 36) were purchased from Charles River post-weaning (aged 21 postnatal days) and maintained in groups of 3 littermates in individually ventilated cages. Cages contained sawdust, paper bedding, and environmental enrichment. Mice were housed at 20 ± 2 °C under a 12-hour light/12-hour dark photoperiod. They received standard rodent-pelleted chow ad libitum (Special Diets Services, Witham, UK). Age (approximate postnatal days; ~PND) was calculated using the mean age at the start of each procedure. Human-equivalent life stage details are provided in Supplementary Table 2. At the end of the study (~PND 394), mice were humanely culled via cervical dislocation. The work was licensed by the UK Home Office (PBDAFDFB0 and P67C4EBE4) and complied with the guiding principles for the care and use of laboratory animals. Ethical approval was granted by Newcastle University Animal Welfare and Ethics Review Body.

### Cranial-Irradiation With PFB

We used the small animal radiation research platform (SARRP) to precisely deliver MB-like CRT with PFB via computerized tomography imaging guidance.^48^ Irradiation was performed at Leeds University. Mice were randomly allocated into 3 treatment groups: cranial (whole-brain) irradiation (“CRT only,” human-equivalent 37.5 Gy EQD2; *n* = 12), cranial-irradiation with an additional PFB (“CRT+PFB,” human-equivalent 37.5 Gy EQD2 with 11.25 Gy EQD2 [48.75 Gy EQD2 to the posterior fossa]; *n* = 12) and a control group that did not receive any irradiation (sham, 0 Gy, *n* = 12). Doses were calculated with an equivalent dose in 2Gy fractions (EQD2) assuming an α/β ratio of 2. Irradiation was delivered at a dose rate of 3.66 Gy/min. For mice in the CRT-only group, treatment began on PND 35 and lasted for 10 days. For the CRT + PFB group, treatment commenced at PND 37 and required an additional 3 days of treatment (details are provided in Supplementary Figure 1). All mice (*n* = 36) including the sham group were anesthetized with isoflurane and placed into the SARRP, independent of receipt of radiation. A 10 × 10 mm collimator with arc from −60 to 60 °C was used to deliver both CRT and the high-dose boost to the posterior fossa. While MB patients receive CSI, here spinal cord irradiation was omitted to limit the development of acute toxicities arising from off-target radiation to the thoracic cavity. Beam angles were selected to avoid the oral cavity, olfactory bulbs, ear, and ear canal. Dose verification was carried out by end-to-end testing by NPL and Innovate UK. This was carried out using 10 × 10 mm and 5 × 5 collimators with both static and arc beams. Differences between alanine pellets and gafchromic film were calculated when exposed to a dose of 12 Gy in a mouse phantom model. The difference between the treatment planning system dose and the measured dose was approximately 5% for film and 3% for pellets. Radiation protocols for each treatment group are summarized in Table 1 and a detailed timeline is provided in Supplementary Figure 1.

**Table 1.** Small Animal Radiation Research Platform Irradiation Regimen

Group	Radiation dose	Radiation schedule	Equivalent radiation dose in 2 Gy fractions (EQD2)
**CRT only**	Whole brain: 10F × 3 Gy (30 Gy)	5 days per week for 2 weeks	Whole-brain: 37.5 Gy
**CRT + PFB**	Posterior fossa only: 3F × 3 Gy (9 Gy) Whole brain: 10F × 3 Gy (30 Gy)	3 consecutive days 5 days per week for 2 weeks	PF only: 11.25 Gy [48.75 total] Whole-brain: 37.5 Gy
**Sham**	*Did not receive radiation*		

Radiation dose and frequency for cranial-radiation (CRT) only, CRT with posterior fossa boost (CRT + PFB), and sham-irradiated control group (sham); *n* = 12 for each group. The total human-equivalent radiation dose per area is given in brackets. While < 2 Gy per fraction is typical for human MB radiotherapy regimens, regulatory limitations necessitated the delivery of 3 Gy per fraction in our model, resulting in an overall dose equivalent to 37.5 Gy EQD2. Anesthesia (isoflurane) was administered to all groups independent of receipt of CRT.

Following recovery, mice were transferred to Newcastle University (~PND 63) for subsequent assessment, where assessors were blind to the allocation of radiation group. After acclimatization, mice received radiofrequency identification (RFID; IMI-500 Read Only Transponder), implanted subcutaneously under general anesthesia. Mice were longitudinally assessed for over one year (up to ~PND 394).

### Frailty Assessment

Frailty assessment was carried out using the Rockwood-style FI as previously described.^49^ Briefly, 30 parameters of frailty (summarized in Supplementary Table 3) were scored on a scale from 0 (no impairment) to 1 (severe impairment). Grip strength was measured using the BIO-GS3 (BIOSEB), and the mean of 3 attempts was calculated from the maximal peak force generated (grams, g) from the forepaws. Body weight, body temperature, and grip strength were scored according to degrees of standard deviation (S.D.) from the mean of age- and sex-matched controls (0: < 1SD; 0.3: 1SD–2SD; 0.7: 2SD–3SD; 1: ≥ 3SD). To minimize subjectivity, assessors (*n* = 2) were kept the same throughout and blind to the allocation of radiation group; however, some unavoidable visual indications of therapy receipt were present.

### Physical Functioning Assessment

#### Grip strength.—

Neuromuscular function was assessed using the Grip Strength Test (BIO-GS3, BIOSEB) on forepaws. Mice were lowered via the tail onto the device, and the maximal peak force was recorded (grams, g). The mean was calculated from 3 attempts.

#### Rotarod.—

To assess balance, co-ordination, and endurance, mice were placed on the rotarod (Roto-Rod Series 8, IITC Life Science), which began to rotate at an initial speed of 4 rpm, and gradually accelerated by 7.2 rpm per minute.^50^ Time on the rod (seconds) was recorded automatically when mice fell from the rotarod. Quiet, low-light conditions were used to minimize stress during testing. Mice were tested 3 times per day, for 2 consecutive days, with approximately 20-minute intervals between trials. The mean was calculated using scores across both days.

### Neurocognitive Assessment

The Y-maze was used to assess working memory.^51^ The maze consisted of 3 arms made of dark gray plastic; each arm was 40 cm long, 5 cm wide, and 10 cm high. Mice were placed in arm A and observed for 8 minutes; arm entry was manually recorded. Quiet, low-light conditions were used to minimize stress during testing. Spontaneous alternation was defined as the frequency of a mouse entering a novel arm of the maze in 3 consecutive entries (eg, A-B-C), divided by the total arm entries, minus 2.

Learning, short- and long-term memory (LTM) was assessed using the Barnes maze (BM) as previously described.^52^ The BM consisted of 20 holes, surrounded by visual cues (square, circle, cross, and triangle). The target hole contained an escape box underneath. Target hole allocation was randomly assigned across visual cues and treatment groups. The maze was thoroughly cleaned and rotated 90° between each mouse and trial to remove potential olfactory cues. Briefly, testing consisted of 4 steps. (1) Day 1: Initial acclimatization—the mouse was placed in an opaque holding chamber for 10 seconds, then gently guided to the target hole. The mouse stayed in the escape box for 2 minutes. (2) Day 1–4: Spatial acquisition (training period)—after 10 seconds in the holding chamber, the mouse attempted to locate the target hole for up to 3 minutes, after which the mouse remained in the escape box for 1 minute. This was repeated for 4 trials per day for 4 days, with approximately 25 minutes between trials for memory consolidation. (3) Day 5: Probe 1 (short-term memory [STM] test)—24 hours after the final day of spatial acquisition, the escape box was removed from the target hole and the mice explored the maze for 90 seconds. 4) Day 12: Probe 2 (LTM test)—7 days after probe 1, the escape box was removed from the target hole and the mice explored the maze for 90 seconds. No training/testing took place on days 6–11. primary latency (PL) was defined as the time taken to locate the target hole.

### Statistical Analysis

Statistical analysis and data visualization were carried out using SPSS statistics (IBM, version 27) and R Studio (version 4.2.2). T-tests (independent and paired), linear regression, and ANOVA with post-hoc Tukey tests were used to compare group means between continuous variables. Significant associations were defined as having a p < 0.05. Where appropriate, the Benjamini-Hochberg procedure was used to correct for multiple tests. Kaplan–Meier curves with log-rank tests were used to visualize survival, and deaths not related to irradiation were right censored (details provided in Supplementary Table 4).

## Results and Discussion

### Development of a Clinically Relevant, High-Dose, Targeted Cranial-Irradiation Model of MB Treatment

While previous in vivo modeling studies each contain critical limitations (a summary is provided in Supplementary Table 1), ours is the only model to (1) deliver fractioned high-dose radiotherapy, (2) use juvenile mice, and (3) perform longitudinal and comprehensive long-term follow-up, making it optimally positioned for use in future interventional development.

Previous studies typically utilized traditional approaches to deliver radiation to the whole head, with lead shielded from the body.^26,27,39,53,54^ Delivery via this modality renders non-target regions such as the mouth, ears, and eyes are in the radiation field. Consequently, whole-head irradiation comes with high levels of acute toxicity, (eg,. damage to the salivary gland, mouth ulceration, eye dryness, and weight loss).^43,44^ Moreover, whole-head irradiation cannot deliver targeted radiation to specific brain regions, which prevents the delivery of a PFB dose. Thus, irradiation using these methodologies cannot fully recapitulate the dose and targeting used in MB regimens.

Childhood MB patients with high-risk disease typically receive 36 Gy CRT with 54 Gy to the posterior fossa; these high doses are associated with the most severe late effects and represent the greatest clinical need.^15,55^ By utilizing a pre-clinical radiotherapy platform (SARRP) to deliver computerized tomography image-guided, arc-delivered, fractionated radiotherapy CRT (Supplementary Figure 1), we were able to deliver up to an equivalent of 48.75 Gy EQD2 to the posterior fossa, very close to the high-dose PFB used in medulloblastoma regiments.^56^

Radiotherapeutic insult to the brain results in substantial damage to healthy tissue, including damage to the vasculature and demyelination, resulting in impaired neurogenesis.^57^ As significant brain development occurs in early childhood, young children are particularly vulnerable to the deleterious consequences of CRT in treating a brain tumor.^58^ We delivered CRT to cohorts of young mice at ~PND 36, equivalent to the juvenile life stage in humans and peak stage of MB diagnosis.^15^

**Figure 1.**
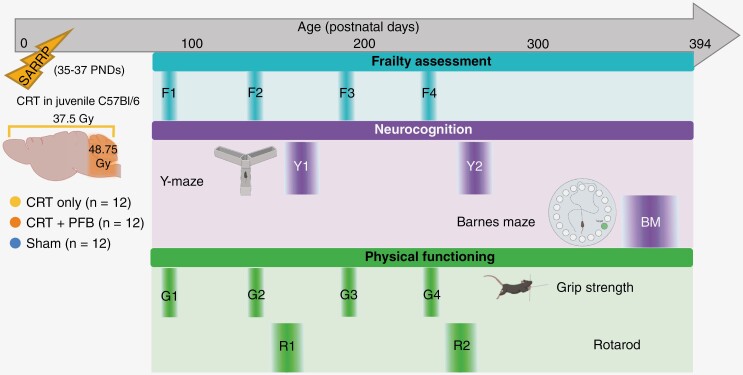
Schematic overview of the development of a clinically relevant, high-dose, targeted cranial-irradiation model. An overview of the study design. Juvenile C57Bl/6J mice (age 35–37 PNDs) received either CRT only (37.5 Gy human equivalent EQD2, *n* = 12), CRT + PFB (total dose of 48.75 Gy EQD2 to the posterior fossa, human-equivalent), or sham-irradiation (*n* = 12) via the small animal radiation research platform. Following irradiation, mice were subjected to longitudinal functional assessments. Assessments of frailty (frailty assessment at 4 timepoints [F1: ~PND 97, F2: ~PND 130, F3:~PND 191 and F4: ~PND 233]), neurocognition (Y-maze at 2 timepoints [Y1: ~PND 179 and Y2: ~PND 266] and Barnes maze [BM: ~PND 369]) and physical functioning (grip strength test at 4 timepoints [G1: ~PND 97, G2: ~PND 130, G3:~PND 191, and G4: ~PND 233] and RotaRod at 2 timepoints 1-2 [R1: ~PND 172 and R2: ~PND 249]) were carried out up to ~PND 394.

While previous studies have assessed the effects of cranial irradiation in young mice, often this is for a relatively short period.^35,38,46^ We followed up with our mouse cohort for over a year (up to ~PND 394); longitudinal assessments of frailty, physical functioning (grip strength and rotarod), and neurocognition (Y maze and BM) were performed to determine their sensitivity to CRT, additional negative consequences of PFB, and the extent to which our model recapitulated MB late-effect severity and durability (Figure 1).

### Our Human-Equivalent CRT Regimen was Well Tolerated, Acutely, and Mice Thrived Post-treatment

**Figure 2.**
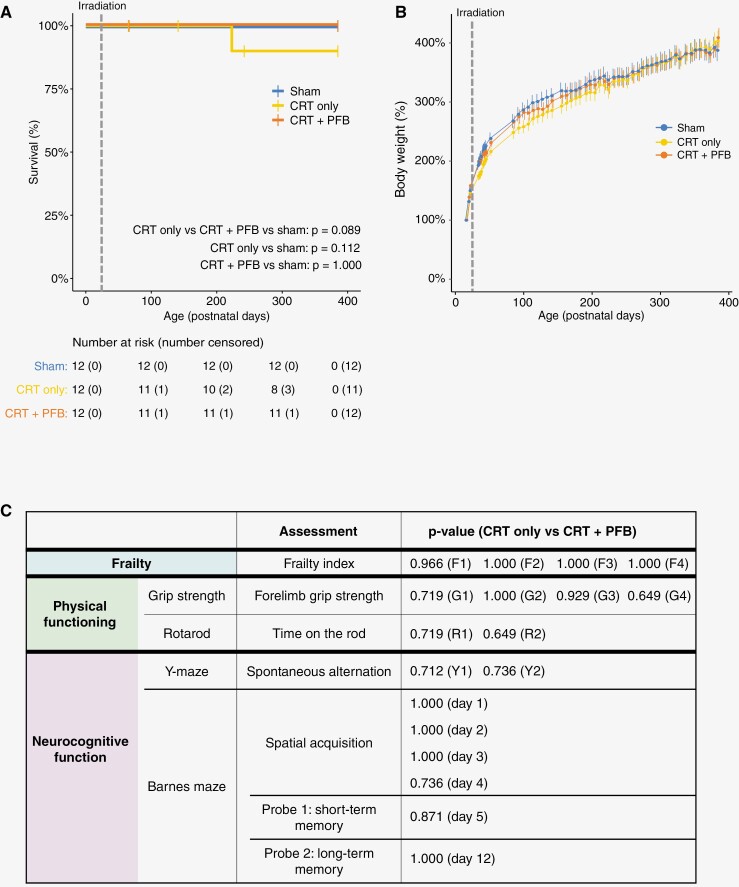
Clinically relevant, high-dose, targeted cranial irradiation is well tolerated and mice thrive independent of posterior fossa boost. (A) Kaplan–Meier plot of survival by cranial-radiation group. Deaths not related to radiation were right-censored (details are provided in Supplementary Table 4) Receipt of cranial irradiation is depicted by the dotted line. (B) Mean body weight (+ SEM) was measured at least weekly over the course of the study, pre- and post-irradiation (*P*-values given in Supplementary Table 5). The receipt of cranial irradiation is depicted by the dotted line. (C) Summary of the performance of CRT only versus CRT + PFB groups. Adjusted *P*-values following independent *t*-tests between CRT only and CRT + PFB for all assessments of frailty, physical functioning (grip strength and Rotarod), and neurocognition (Y-maze and Barnes maze). A full comparison of all measures tested is provided in Supplementary Tables 6A–C).

High-dose CRT was well tolerated; no animals died due to severe acute toxicity either during or immediately following irradiation (Figure 2A). Over the course of the experiment, 5 mice were culled (though not attributable to cranial irradiation; Supplementary Table 4). Following irradiation, mice thrived and continued to grow at the same rate as sham-irradiated controls, independent of receipt of PFB (Figure 2B, *P*-values shown in Supplementary Table 5), throughout the life course.

Outcomes for the CRT + PFB group were equivalent to the CRT group across the vast majority of measures (*n* = 163/166) of frailty, physical functioning, and neurocognition tested, indicating there was no additional impact of the PFB dose (Figure 2C and Supplementary Tables 6A–C). Previous studies have shown that the dose of cranial irradiation is the major driver of poor neuropsychological outcomes in children treated for posterior fossa tumours^55^ and others have reported a selective vulnerability of specific neuro-anatomical substructures to radiation injury, implicating the hippocampus and frontal- and temporal lobes in determining neurocognitive function, rather than the posterior fossa,^59–61^ supporting our findings. Given that PFB is not associated with late-effect severity in our model, CRT only and CRT + PFB groups were subsequently combined for further analyses (henceforth collectively referred to as CRT) for comparison against sham-irradiated control mice.

### CRT Drives Accelerated Frailty

**Figure 3.**
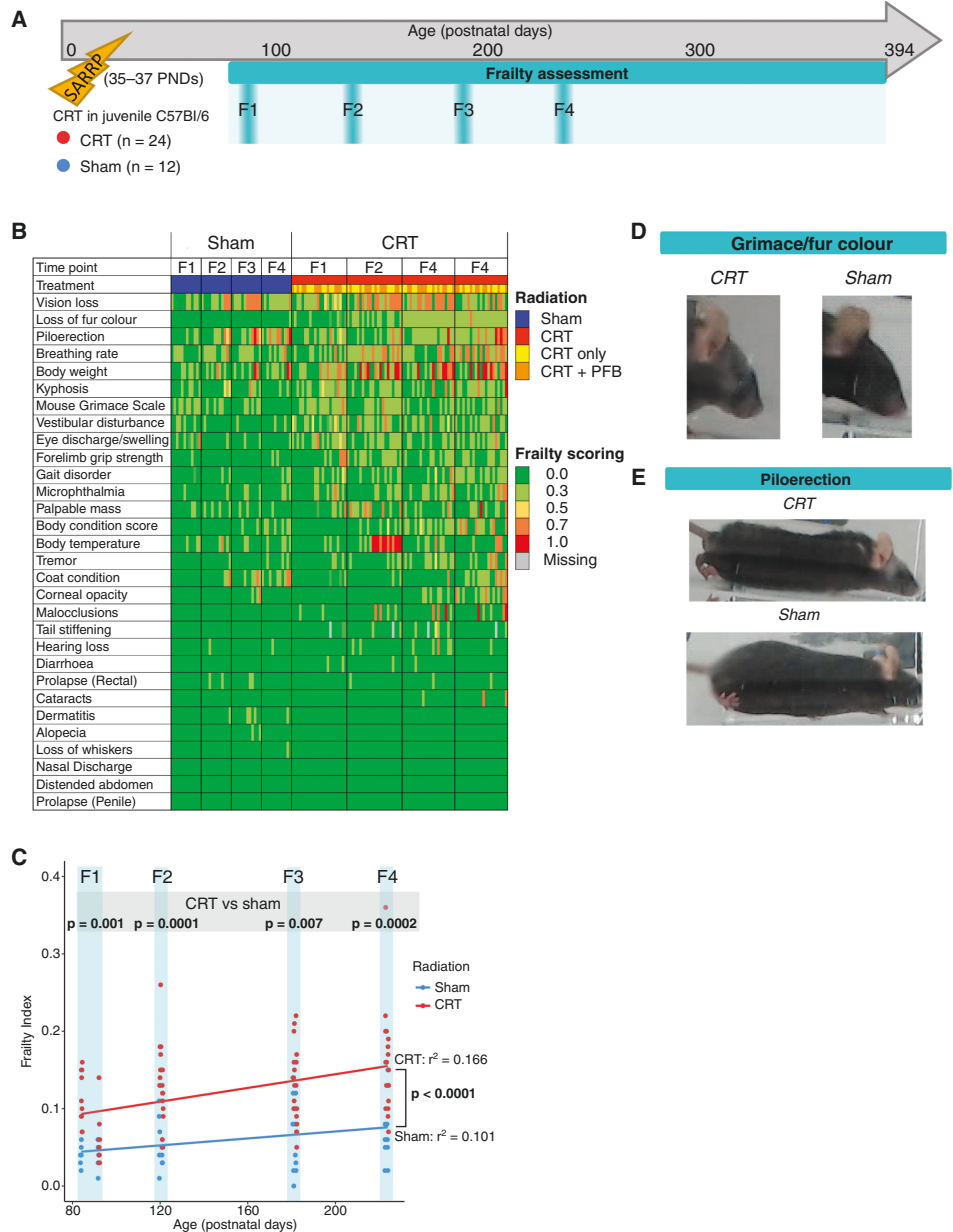
CRT drives accelerated development of frailty. (A) Timeline of longitudinal frailty assessment (F1: ~PND 97, F2: ~PND 130, F3: ~PND 191 and F4: ~PND 233). (B) Increased frailty scores following CRT. Heatmap showing frailty scores for all 30 frailty parameters following CRT or sham-irradiation. Individual frailty criteria were scored from 0 (no impairment, green) to 1 (severe frailty, red). Gray shading depicts missing data. Criterion are ordered from most commonly impaired to least commonly impaired (top to bottom). (C) CRT drives accelerated frailty. Mean frailty index (FI) following longitudinal frailty assessment at F1-4. Each point represents individual mice scores for CRT (red) and sham (blue) groups. Rate of frailty increase is higher in CRT-treated mice. Goodness of fit is denoted by r^2^, *P*-value represents linear regression. Significant *P*-values (*P* < .05) are in bold text. Examples of commonly impaired features following CRT or sham-irradiation: Are grimace and loss of fur color following CRT (D) and piloerection following CRT (E).

Survivors of childhood MB experience increased frailty.^2,14,62^ To assess whether CRT induced frailty in our model, we used the frailty index (FI), a measure used to provide an overall picture of general health and well-being in both mice and humans. The FI is also predictive of mortality across species.^63,64^ The mouse FI exhibits key features of the FI used in humans and is therefore useful to quantify deficits relevant to human frailty and aging. The FI can be influenced by stress; however, in our study, this was minimized by acclimatization to handling prior to assessments and maintaining low levels of environmental noise. Frailty was assessed longitudinally (at ~PND 97, 130, 191, and 233; time points F1-4, respectively, Figure 3A) in our mice by scoring 30 age-related conditions on a scale of 0 (no frailty) to 1 (severe frailty), and calculating an average to produce a FI as described (Figure 3B).^49,65^

As expected, the FI for sham-irradiated mice increased along over the life course (r^2^ = 0.101), representing a normal, healthy, aging profile.^64^ However, CRT-induced accelerated frailty manifested early; at F1 (~PND 97; human-equivalent of early adulthood) FI was significantly higher in the CRT group than the sham-irradiated group (*P* = .001) and this persisted at all time points (F2, *P* = .0001; F3, *P* = .007 and F4, *P* = .002, Figure 3C). The rate of FI increase was significantly higher following CRT (r^2^ = 0.166, *P* < .0001; Figure 3C). Vision loss, loss of fur color, piloerection, and high breathing rate were the most common FI parameters following CRT (Figure 3B; representative images are shown in Figure 3D and E). FI scores for distinct CRT only and CRT + PFB groups are summarized in supplementary figure 3.

### Physical Functioning is Impaired Following Cranial-Irradiation

**Figure 4.**
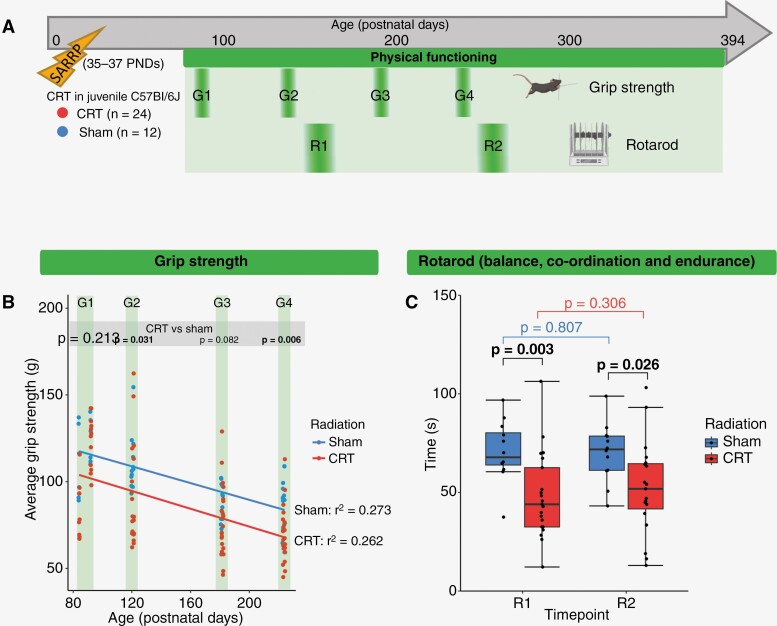
Physical functioning is impaired following cranial irradiation. (A) Timeline of longitudinal physical functioning assessment via the Grip Strength test (G1: ~PND 97, G2: ~PND 130, G3: ~PND 191 and G4: ~PND 233), and the RotaRod (R1: ~PND 172 and R2: ~PND 249). (B) Grip strength was poorer following CRT than sham-irradiation. Scatterplot showing longitudinal grip strength (mean of 3 attempts) at G1-4, where each point represents individual mice for CRT (red) and sham (blue) groups with linear regression fit lines. Significance was assessed via independent *t*-tests. Grip strength declines over time. Goodness of fit is denoted by r^2^. (C) Balance, coordination, and endurance are worse following CRT than sham-irradiation. Average time on the Rotarod (mean time across 6 trials) at R1 and R2. Each point represents individual mice. Significance was assessed via independent *t*-tests (black [at both R1 and R2]) and paired *t*-tests (red [R1 vs. R2 in the CRT group] and blue [R1 vs. R2 in the sham-irradiation group]). Significant *P*-values (*P* < .05) are in bold text.

Physical functioning was assessed longitudinally using the grip strength test (at ~PND 97, 130, 191, and 233; time points G1-4, respectively) and rotarod (~PND 172 and 249; time points R1-2, respectively, Figure 4A). The grip strength of the CRT group was worse than sham-irradiated mice at all time points; this was significant at G2 (~PND 130, *P* = .031) and G4 (~PND 233, *P* = .006; Figure 4B). Both CRT and sham-irradiated groups exhibited a similar reduction in grip strength with age (Figure 4B).

To assess endurance, and neurological function related to balance and coordination, the mice were subjected to the rotarod at ~PND 172 and ~PND 249 (time points R1 and R2, respectively, Figure 4C). The CRT group were able to stay on the rotarod for significantly less time at both R1 (mean time 48.2 vs. 70.7 s, *P* = .003) and R2 (52.3 vs. 70.2 s, *P* = .026, Figure 4C). There was no age-associated decline in rotarod performance either group (Figure 4C). Physical functioning for distinct CRT only and CRT + PFB groups are summarized in Supplementary Figure 4.

The impaired physical functioning induced by CRT in our model mirrors that of childhood brain tumor survivors. Many survivors of childhood medulloblastoma experience below-average physical functioning, particularly within motor functioning, exhibiting difficulties such as ataxia and coordination disorders, as well as reduced fine motor skills.^10,11,66,67^ While impaired physical functioning may be a result of the tumor itself, younger age at diagnosis and combination treatment approaches including surgical resection, and the use of chemoradiation have been identified as risk factors for neurological dysfunction.^67^

### CRT Induces Deficits in Memory and Learning

**Figure 5.**
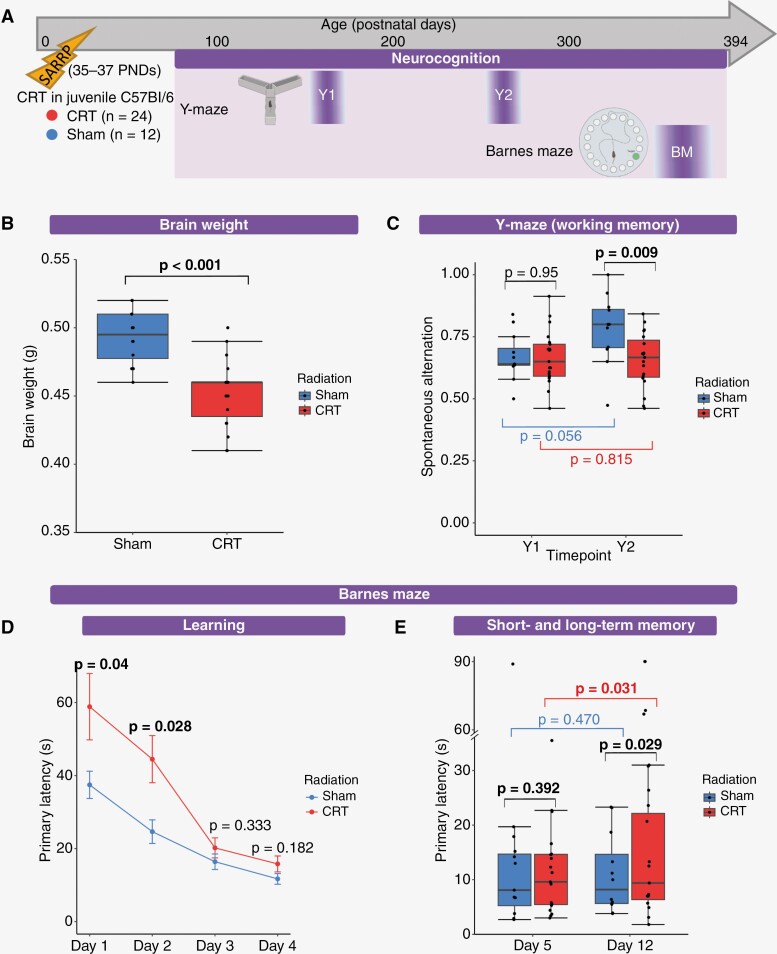
CRT induces deficits in memory and learning. (A) Timeline of longitudinal neurocognitive assessment using the Y-maze (working memory [spontaneous alternation] at Y1: ~PND 179 and Y2: ~PND 266) and the Barnes maze (learning, short- and long-term memory [BM: ~PND 369]). (B) Brain weight is lower following CRT than sham-irradiation. Brain weight (g) at ~PND 394, where each point represents individual mice. Significance was assessed via independent *t*-test. (C) Working memory is poorer following CRT than sham-irradiation. Spontaneous alternation at Y1 and Y2, where each point represents individual mice. Significance was assessed via independent *t*-test (black [at both Y1 and Y2]) and paired *t*-tests (red [Y1 vs. Y2 in CRT group] and blue [Y1 vs. Y2 in sham-irradiation group]). (D) Mice receiving CRT showed initial learning deficits but overcame this by day 3. Mean time is taken to find the target hole (primary latency, s) during spatial acquisition (days 1–4, 4 trials per day). Significance was assessed via independent *t*-tests. (E) Following CRT mice had deficits in long-term memory but not short-term memory. Primary latency on day 5 (short-term memory, STM) and day 12 (long-term memory, LTM), where each point represents individual mice. Significance was assessed via independent *t*-tests (black [at both days 5 and 12]) and paired *t*-tests (red [day 5 vs. 12 in CRT group] and blue [days 5 vs. 12 in sham-irradiation group]). Significant *P*-values (*P* < .05) are in bold text.

Neurocognitive impairment is common in childhood MB survivors.^7,55^ Neurocognitive function was assessed by the Y-maze (at ~PND 179 and ~PND 266; time points Y1 and Y2, respectively) and BM (at ~PND 369; time point BM), and brain weight was measured at the study endpoint (~PND 394; Figure 5A). CRT has been shown to reduce brain volume in both humans and mice,^68^ which has also been linked to lower IQ scores in childhood MB survivors.^69^ In our clinically relevant model, CRT significantly reduced brain size; brain weight was significantly less in the CRT group than in sham-irradiated controls (median weight: 0.46 and 0.50 g, respectively, *P* < .001; Figure 5B). CRT has been linked with decreased total brain volume, decreased white matter, and reduced neurogenesis; however, further investigation into the specific substructure vulnerabilities is required.^68,70^

Neurocognitive function was assessed by the Y-maze (spontaneous alternation) and BM (PL). To assess working memory, mice were subjected to the Y-maze at ~PND 179 and ~PND 266 (time points Y1 and Y2, respectively, Figure 5A). CRT-induced working memory deficits (Figure 5C). While at Y1, early in the life course, the CRT group performed equivalently to the sham-irradiated group (*P* = .95), at Y2 (~PND 266; human-equivalent of early middle age) spontaneous alternation was significantly lower than sham-irradiated mice (mean spontaneous alternation: 0.655 vs. 0.781, *P* = .009, Figure 5C).

The ability to learn and retain learned behavior was assessed using the BM at ~PND 369 (human-equivalent of middle age; Figure 5A). Mice were trained for 4 days to locate the target hole (spatial acquisition [days 1–4], 4 trials per day); search strategy and time to locate the target hole improved following training (as shown in the pre- and post- training video [online resource 1 and 2]). Mice in the CRT group were initially slower to locate the target hole, presumably in part attributable to their worse physical functioning; at days 1 and 2 PL (time to locate target hole) was significantly higher in the CRT group than the sham-irradiated group (day 1 PL: 58.9 vs. 37.5 s [*P* = .04] and day 2 PL: 44.5 vs. 24.6 s [*P* = .028]). However, the CRT group performed equivalently to the sham-irradiated controls by the end of the spatial acquisition period; at day 3 and day 4 PL was equivalent in the CRT and sham-irradiated groups (*P* = .333 and *P* = .182, respectively, Figure 5D). Neurocognitive function for distinct CRT only and CRT + PFB groups are summarized in Supplementary Figure 5.

CRT-induced deficits in LTM but not STM (Figure 5E). A probe trial to assess STM function was performed one day after the spatial acquisition period (day 5). The CRT group showed no deficit in STM; PL was equivalent in CRT and sham-irradiated groups (*P* = .392). After 1 week, with no further training, a second probe trial was conducted to assess LTM (day 12). Despite having performed equally at day 5, mice that received CRT had impaired LTM and took significantly longer to locate the target hole; PL was significantly higher for the CRT group than sham-irradiated controls (day 12 mean PL 27.4 vs. 10.9 s, *P* = .029, Figure 5E). LTM impairment following CRT is analogous to human MB survivors, where the majority of survivors report memory problems.^71^

The neurocognitive deficits induced by CRT in our model mirror those seen in childhood MB survivors. Reduced attention, slower processing speeds, and poor working memory are characteristic of medulloblastoma patients who have received cranial radiotherapy.^21,69,72–77^ Such domains support the acquisition of new learning such that childhood brain tumor survivors acquire new information at half the rate of unaffected peers.^16^ This cranial radiation-induced neurocognitive impairment was mirrored within our in vivo model system; following CRT mice displayed reduced working memory and LTM function. 70%–90% of childhood brain tumor survivors demonstrate significant impairment in global intellectual functioning,^20,21^ which is in turn strongly correlated with decreased quality of life.^22,23,25,77^

Receipt of CRT, particularly at a young age, results in a wide range of deleterious late effects that can drastically reduce the quality of life of childhood cancer survivors.^11,16,71,78^ We conclude that delivery of childhood MB-equivalent radiotherapy is tolerated in vivo and following longitudinal, multi-parameter assessments, invokes an equivalent late-effect profile to the human disease. Thus, our clinically relevant model provides an essential platform that will both facilitate the elucidation of novel/target mechanisms underpinning MB late effects and the development of novel neuro-interventional strategies to alleviate the burden of surviving childhood MB.

## Supplementary Material

vdae091_suppl_Supplementary_Material

## Data Availability

The data that support the study’s findings are available from the corresponding author (DH) upon reasonable request.
