# Supplementary material for: In vivo modeling recapitulates radiotherapy delivery and late-effect profile for childhood medulloblastoma
Source: Neurooncol Adv. 2024 Jun 6;6(1):vdae091. doi: 10.1093/noajnl/vdae091 (PMC11212071; doi:10.1093/noajnl/vdae091)
Supplement: vdae091_suppl_Supplementary_Material [file vdae091_suppl_supplementary_material.pdf]

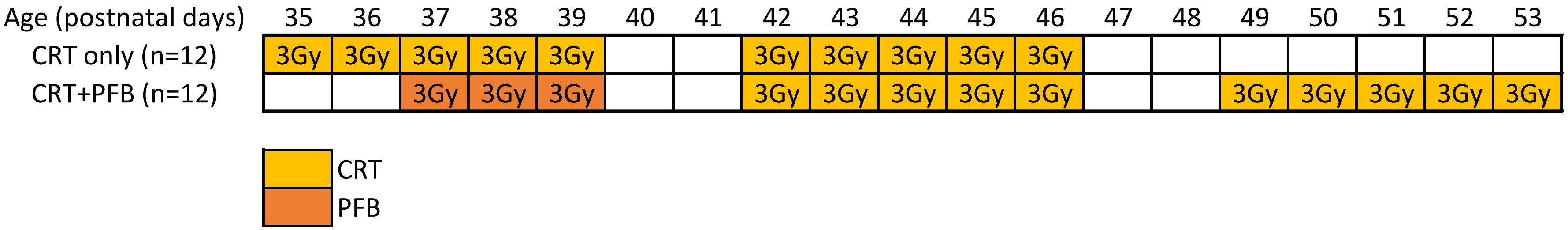

Supplementary figure 1. Timeline of radiation delivery. Mice in the CRT only group received 10 fractions of 3 Gy radiation to the whole-brain (yellow shading) starting at PND 35. Mice in the CRT+PFB group received a total of 13 fractions of 3 Gy radiation starting at PND 37. A posterior fossa boost (PFB; orange shading) consisting of 3 fractions of 3 Gy radiation was delivered to the posterior fossa only, followed by CRT (10 Fractions of 3 Gy to the whole-brain).

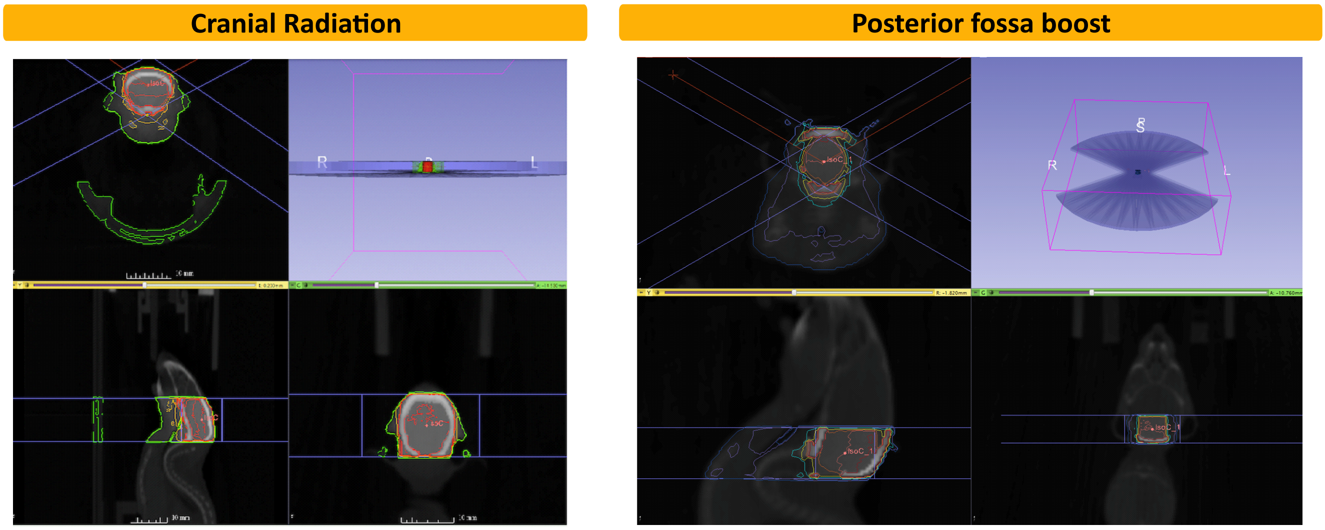

Supplementary figure 2. A representative image of dose distribution in each of the 3 planes for delivery of whole-brain radiation (CRT; left) and a posterior fossa boost (PFB; right) using the SARRP in a young mouse, using 2x 60 degree arc treatments and CT guidance. IsoC = isocentre. High dose volume shown by red outline.

A.

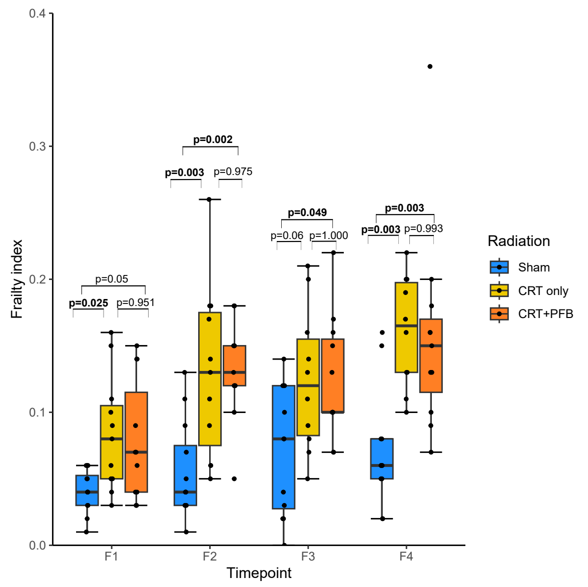

B.

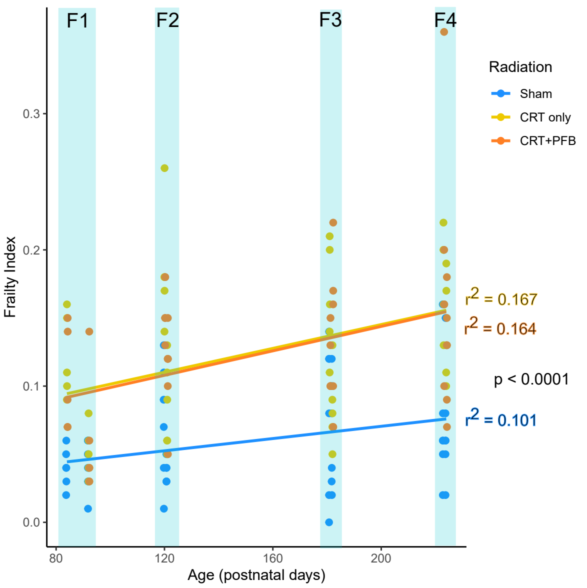

C.

	Sham			CRT only			CRT+PFB		
	F2	F3	F4	F2	F3	F4	F2	F3	F4
F1	0.155	0.053	**0.02**	**0.001**	**0.006**	**0.001**	**0.003**	**0.02**	**0.005**
F2	-	0.23	0.311	-	0.317	0.143	-	0.636	0.338
F3	-	-	0.97	-	-	**0.029**	-	-	0.163

Supplementary figure 3. Cranial-irradiation drives accelerated frailty, independent of receipt of PFB. A) CRT drives accelerated frailty, independent of receipt of PFB. Longitudinal FI (mean frailty score across all 30 criteria) at F1-4, where each point represents individual mice. Significance tested via one-way ANOVA with post-hoc Tukey, p<0.05 are bold. B) Frailty increases faster in CRT than sham-irradiation, independent of receipt of PFB. Scatterplot showing FI for each mouse, with linear regression lines. C) p-values following paired-t tests between all longitudinal frailty assessment points (F1-4), p<0.05 are in bold.

Grip strength

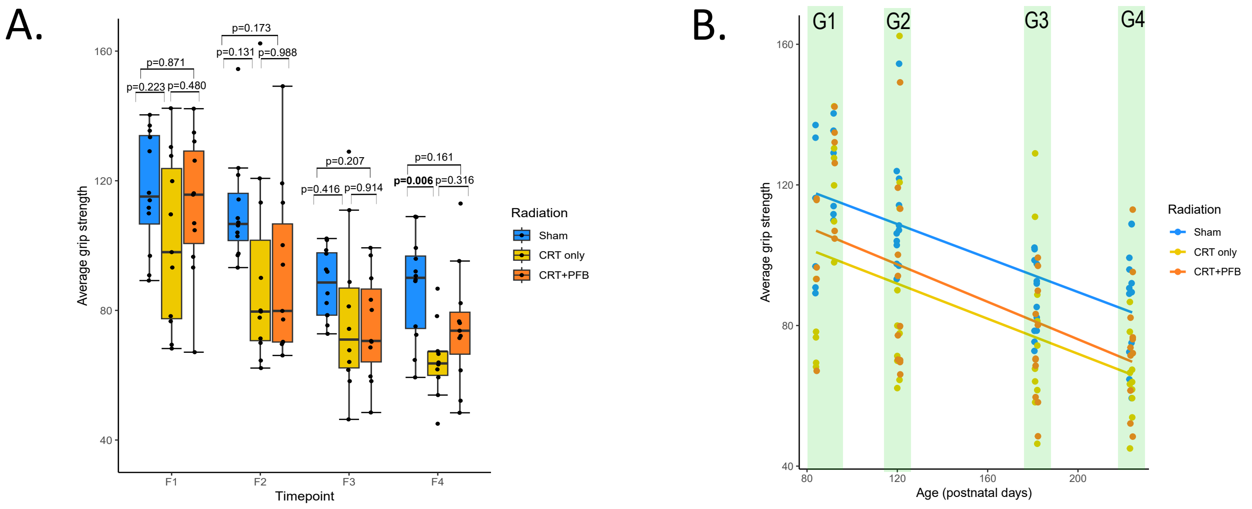

C.

	Sham			CRT			CRT+PFB		
	G2	G3	G4	G2	G3	G4	G2	G3	G4
G1	0.346	**0.0003**	**0.0004**	**<0.0001**	0.112	**0.006**	**0.035**	**0.002**	**0.001**
G2	-	**0.0003**	**0.0001**	-	0.315	**0.033**	-	0.135	0.125
G3	-	-	0.84	-	-	**0.038**	-	-	0.96

Rotarod (balance, co-ordination and endurance)

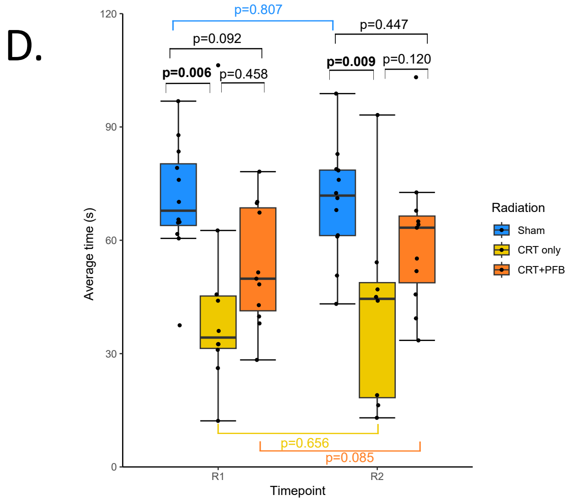

Supplementary figure 4. Physical functioning is impaired following cranial irradiation, independent of PFB. A) Grip strength was poorer following CRT than sham-irradiation. Longitudinal grip strength (mean of 3 attempts) at G1-4, where each point represents individual mice. Significance was tested via one-way ANOVA with post-hoc Tukey, $p<0.05$ are bold.. B) Grip strength declines over time. Scatterplot showing mean grip strength for each mouse, with linear regression lines. C) p-values following paired-t tests between all longitudinal grip strength at assessment points (G1-4), $p<0.05$ are in bold. D) Balance, co-ordination and endurance is worse following CRT than sham-irradiation, independent of receipt of PFB. Average time on Rotarod (mean time across six trials) at R1 and R2. Each point represents individual mice. Significance assessed via independent t-tests (black [at both R1 and R2]) and paired t-tests (blue [R1 vs R2 in sham-irradiation group], yellow [R1 vs R2 in CRT only group] and orange [R1 vs R2 in CRT+PFB group]), $p<0.05$ are bold.

Y-maze (working memory)

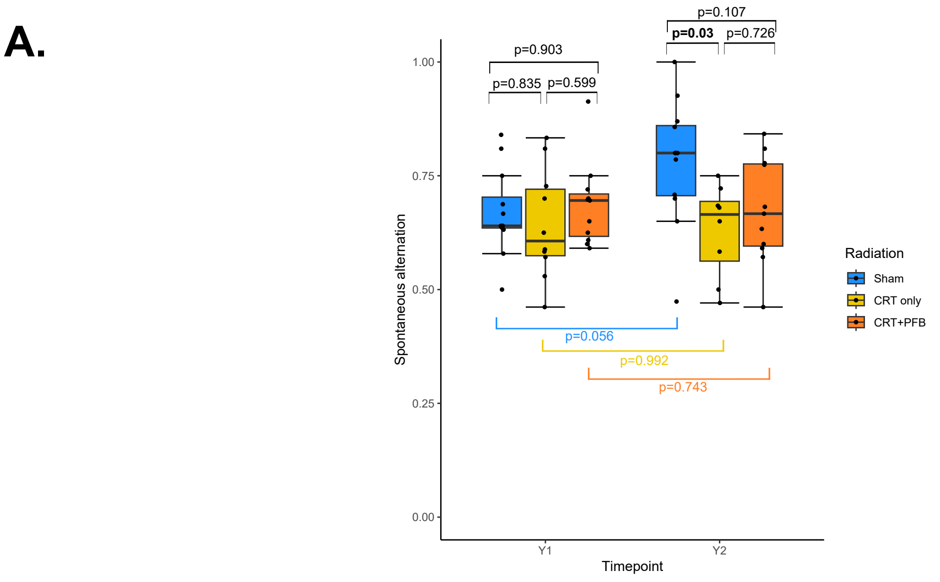

Barnes maze (learning, short- and long-term memory)

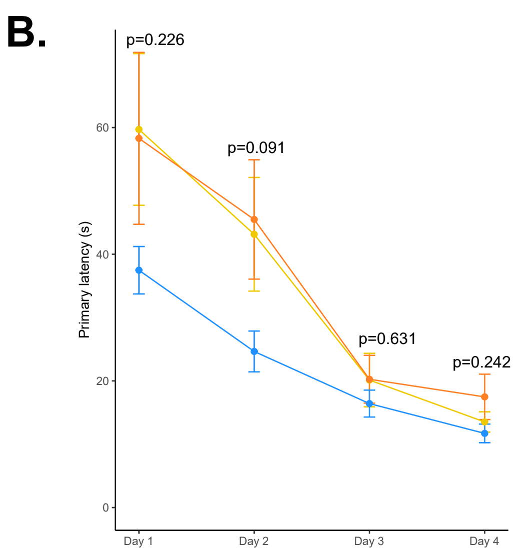

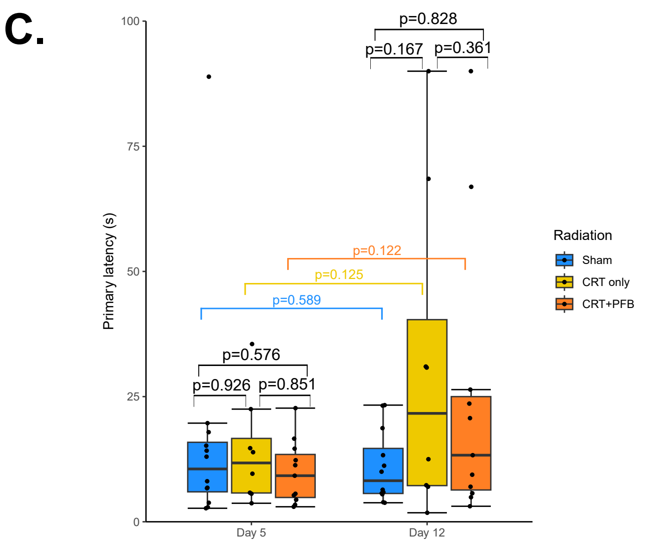

Supplementary figure 5. Cranial irradiation induces deficits in memory and learning, independent of receipt of PFB. A) Working memory is poorer following CRT than sham-irradiation. Spontaneous alternation (SA) at Y1 and Y2, where each point represents individual mice. Significance was assessed via independent t-tests (black [at both Y1 and Y2]) and paired t-tests (blue [Y1 vs Y2 in sham-irradiation group], yellow [Y1 vs Y2 in CRT only group] and orange [Y1 vs Y2 in CRT+PFB group]), and bold when $p<0.05$. B) Learning during spatial acquisition training of the Barnes maze. Mean time taken to find the target hole (primary latency) during spatial acquisition (day 1-4, four trials per day). Significance tested via one-way ANOVA, $p<0.05$ are bold. C) Primary latency on day 5 (short-term memory, STM) and day 12 (long-term memory, LTM), where each point represents individual mice. Significance was assessed via independent t-tests (black [at both day 5 and day 12]) and paired t-tests (blue [day 5 vs day 12 in sham-irradiation group], yellow [day 5 vs day 12 in CRT only group] and orange [day 5 vs day 12 in CRT+PFB group]), p is bold when $p<0.05$.

Supplementary table 1. A summary of the *in vivo* radiotherapy literature in terms of clinical relevance to paediatric brain tumours (dose, developmental stage, targeting, late-effect assessment and follow up). The studies are summarised according to the following criteria:) Use of a clinically-relevant radiation dose (single or cumulative) of 18 Gy or higher to the brain, ii) use of juvenile mice under PND 42, iii) delivery of targeted radiation to the brain or sub-region of the brain, iv) comprehensive assessment of the late-effect profile (>2 types of assessments [e.g. neurocognition, physical function, frailty]), v) long-term follow up (≥ 6 months post-irradiation).

Study	Clinically-relevant MB radiation dose	Juvenile	Targeted cranial irradiation	Assessment of late-effect profile	Long-term follow up	Reference
Fielder *et al*	X	X	X	✓	✓	44
Tang *et al*	✓	✓	X	X	✓	36
Yuen *et al*	X	✓	X	X	X	31
Sandor *et al*	X	✓	X	X	X	35
Rao *et al*	✓	✓	X	✓	X	32
Moravan *et al*	✓	X	X	X	X	56
de Guzman *et al*	X	✓	X	X	X	49
Ungvari *et al*	✓	X	X	✓	✓	25
Yabluchanskiy *et al*	✓	X	X	X	✓	37
Wong-Goodrich *et al*	X	X	X	✓	X	26
Tomé *et al*	X	X	✓	✓	X	41
Belcher *et al*	✓	X	✓	X	X	28
Baumann *et al*	✓	X	✓	X	X	29
Suckert *et al*	✓	X	✓	X	X	27
Lazarini *et al*	X	X	✓	X	✓	30
Beera *et al*	X	✓	✓	X	X	34
Ruddy *et al*	X	✓	X	X	X	33
Zhang *et al*	X	✓	✓	X	X	39
Zhou *et al*	X	✓	X	X	X	40
Casciati *et al*	X	✓	X	X	✓	58
Zanni *et al*	X	✓	X	X	X	38
Castle *et al*	✓	✓	✓	✓	✓	This manuscript

Supplementary table 2. Mouse and human equivalent life-stage.

Timepoint	Mouse age (PNDs)	Life-stage equivalent
**CRT**	35-37	Juvenile
**F1/G1**	97	Early adulthood
**F2/G2**	130	Early adulthood
**F3/G3**	191	Early adulthood
**F4/G4**	233	Early middle age
**R1**	172	Early adulthood
**R2**	249	Early middle age
**Y1**	179	Early adulthood
**Y2**	266	Early middle age
**BM**	369	Middle age

Supplementary table 3. Parameters assessed during assessment of Frailty. Taken from Whitehead et al

System and Parameter	Potential Deficits
Integument	
Alopecia	Hair loss due to age-related balding and/or barbering (fur trimming)
Loss of fur colour	Change in fur colour from black to grey
Dermatitis	Inflammation, over-grooming, barbering or scratching causing skin erosion. Can result in open sores anywhere on the body
Loss of whiskers	Loss of vibrissae (whiskers) due to aging and/or whisker trimming
Coat condition	Ruffled fur and/or matted fur. Ungroomed appearance. Coat does not look smooth, sleek, and shiny
Physical/musculoskeletal	
Tumours/masses	Development of tumours or masses anywhere on the body
Distended abdomen	Enlarged abdomen. May be due to tumour growth, organ enlargement, or intraperitoneal fluid accumulation
Kyphosis	Exaggerated outward curvature of the lower cervical/thoracic vertebral column. Hunched back or posture
Tail stiffening	Tail appears stiff, even when animal is moving in the cage. Tail does not wrap freely when stroked
Gait disorders	Lack of coordination in movement including hopping, wobbling, or uncoordinated gait. Wide stance. Circling or weakness
Tremor	Involuntary shaking at rest or during movement
Forelimb grip strength	A decline in forelimb grip strength
Body condition score	Visual signs of muscle wasting or obesity based on the amount of flesh covering bony protuberances
Vestibulocochlear/auditory	
Vestibular disturbance	Disruption in the ability to perceive motion and gravity. Reflected in problems with balance, orientation, and acceleration
Hearing loss	Failure to respond to sudden sound (e.g., clicker) indicative of hearing loss or impairment
Ocular/nasal	
Cataracts	Clouding of the lens of the eye. An opaque spot in the centre of the eye
Corneal opacity	Development of white spots on the cornea. Cloudy cornea
Eye discharge/swelling	Eyes are swollen or bulging (exophthalmia). They may exhibit abnormal secretions and/or crusting
Microphthalmia	Eyes are small and/or sunken. May involve one or both eyes
Vision loss	Vision loss, indicated by failure to reach toward the ground when lowered by the tail
Nasal discharge	Signs of abnormal discharge from the nose
Digestive/urogenital	
Malocclusions	Incisor teeth are uneven or overgrown. Top teeth grow back into the roof of the mouth or bottom teeth are long and easily seen
Rectal prolapse	Protrusion of the rectum just below the tail
Penile prolapse	Penis cannot re-enter the penile sheath.
Diarrhoea	Faeces on the walls of the home cage. Bedding adheres to faeces in cage. Faeces, blood, or bedding around the rectum
Respiratory	
Breathing rate/depth	Difficulty breathing (dyspnoea), pulmonary congestion (rales), and/or rapid breathing (tachypnoea)
Discomfort	
Mouse Grimace Scale	Measure of pain/discomfort based on facial expression. Assessment of five facial features: orbital tightening, nose bulge, cheek bulge, ear position (drawn back), or whisker change (either backward or forward)
Piloerection	Involuntary bristling of the fur due to sympathetic nervous system activation
Other	
Temperature	Increase or decrease in body temperature
Weight	Increase or decrease in body weight

Supplementary table 4. Deaths not related to irradiation. Deaths as a result of injury (fighting), procedure or weight loss were right-censored in the survival analysis (figure 2A).

Cause of death	n	Treatment group	Age (Postnatal day)
Injury (fighting)	1	CRT only	74
Injury (fighting)	1	CRT+PFB	75
Procedure	1	CRT only	150
Weight loss	1	CRT only	251

Supplementary table 5. Comparison of body weights relative to start weight for CRT only and CRT+PFB vs sham-irradiation. Adjusted p-values following independent t-tests.

Day	P (CRT only vs sham)	P (CRT+PFB vs sham)
28	0.989	0.983
30	0.989	0.983
42	0.563	0.983
43	0.489	0.983
44	0.489	0.983
45	0.489	0.983
46	0.489	0.983
49	0.489	0.983
50	0.489	0.983
51	0.489	0.983
52	0.489	0.983
53	0.489	0.983
60	0.489	0.983
93	0.670	0.983
101	0.563	0.983
108	0.489	0.983
115	0.517	0.983
122	0.590	0.983
129	0.621	0.983
136	0.670	0.983
143	0.670	0.983
150	0.670	0.983
164	0.670	0.983
171	0.783	0.983
178	0.889	0.983
185	0.935	0.983
191	0.935	0.983
200	0.935	0.983
206	0.935	0.983
213	0.889	0.983
220	0.989	1.000
227	0.948	0.983
233	0.989	0.983
241	0.989	0.983
248	0.989	0.983
254	0.989	0.983
261	0.989	0.983
269	0.989	0.983
277	0.989	0.983
283	0.989	0.983
290	0.989	0.983
297	0.989	0.983
304	0.989	0.983
311	0.989	0.983
317	0.989	0.983
324	0.989	0.983
332	0.989	0.983
338	0.989	0.983
347	0.989	0.983
355	0.989	0.983
361	0.989	0.983
368	0.989	0.983
376	0.989	0.983
382	0.989	0.983
389	0.989	0.983
394	0.989	0.983

Supplementary table 6. Assessment of the additional impact of PFB. Adjusted p-values following independent t-tests between CRT only and CRT+PFB for all assessments of frailty (Supplementary table 6A), physical function (assessed via grip strength and Rotarod, Supplementary table 6B), and neurocognitive function (assessed via Y-maze and Barnes maze, Supplementary table 6C). p-values <0.05 are in bold, green shading represents a worse performance of the CRT+PFB only group and red shading represents a worse performance of the CRT only group.

Supplementary table 6A.

Assessment	*F1*	*F2*	*F3*	*F4*
Average frailty score	0.082 vs 0.0771 (p=0.966)	0.128 vs 0.133 (p=1.000)	0.123 vs 0.124 (p=1.000)	0.1597 vs 0.157 (p=1.000)
Body weight (score)	0.23 vs 0.34 (p=0.719)	0.291 vs 0.609 (p=0.650)	0.26 vs 0.573 (p=0.650)	0.33 vs 0.573 (p=0.652)
Body weight (g)	27.7 vs 26.8 (p=0.736)	30.636 vs 28.609 (p=0.650)	34.67 vs 32.282 (p=0.650)	36.74 vs 32.427 (p=0.626)
Loss of whiskers	0 vs 0	0 vs 0	0 vs 0	0 vs 0
Loss of fur colour	0.082 vs 0.041 (p=0.736)	**0.3 vs 0.055 (p=0.004)**	0.3 vs 0.3 (p=1.000)	0.3 vs 0.336 (p=0.719)
Coat condit on	0.041 vs 0.014 (p=0.736)	0.055 vs 0 (p=0.650)	0.07 vs 0 (p=0.720)	0.32 vs 0.173 (p=0.650)
Alopecia	0 vs 0	0 vs 0	0 vs 0	0 vs 0
Piloerect on	0.23 vs 0.16 (p=0.736)	0.236 vs 0.109 (p=0.652)	0.34 vs 0.218 (p=0.652)	0.48 vs 0.418 (p=0.851)
Dermat t s	0 vs 0	0 vs 0	0 vs 0	0 vs 0
Breathing rate	0.1636 vs 0.1182 (p=0.845)	0.382 vs 0.409 (p=0.946)	0.23 vs 0.236 (p=1.000)	**0.66 vs 0.227 (p=0.003)**
Mouse Grimace Scale	0.282 vs 0.191 (p=0.677)	0.164 vs 0.227 (p=0.762)	0.12 vs 0.109 (p=1.000)	0.15 vs 0.2 (p=0.835)
Cataracts	0 vs 0	0 vs 0	0 vs 0.027 (p=0.720)	0 vs 0.127 (p=0.650)
Corneal opacity	0 vs 0	0 vs 0	0.09 vs 0.082 (p=1.000)	0.16 vs 0.327 (p=0.650)
Microphthalmia	0.055 vs 0.173 (p=0.649)	0.109 vs 0.082 (p=0.871)	0.12 vs 0.2 (p=0.719)	0.19 vs 0.182 (p=1.000)
Eye discharge/swelling	0.1364 vs 0.3 (p=0.649)	0.164 vs 0.164 (p=1.000)	0.06 vs 0.164 (p=0.650)	0.16 vs 0.218 (p=0.834)
Nasal Discharge	0 vs 0	0 vs 0	0 vs 0	0 vs 0
Kyphosis	0.2364 vs 0.2136 (p=0.949)	0.164 vs 0.164 (p=1.000)	0.06 vs 0.245 (p=0.650)	0.21 vs 0.318 (p=0.719)
Tail st f ening	0.03 vs 0 (p=0.649)	0.06 vs 0 (p=0.946)	0.122 vs 0.027 (p=0.665)	0 vs 0.027 (p=0.736)
Vest bular disturbance	0.1955 vs 0.1864 (p=1.000)	0.227 vs 0.2 (p=0.645)	0.24 vs 0.109 (p=0.650)	0.19 vs 0.164 (p=0.946)
Vision loss	0.182 vs 0.291 (p=0.677)	0.464 vs 0.464 (p=1.000)	0.51 vs 0.5 (p=1.000)	0.4 vs 0.173 (p=0.649)
Gait disorder	0.1273 vs 0.0818 (p=0.835)	0.109 vs 0.082 (p=0.871)	0.15 vs 0.055 (p=0.720)	0.24 vs 0.191 (p=0.726)
Tremor	0.055 vs 0.027 (p=0.835)	0.082 vs 0.055 (p=0.871)	0.2 vs 0.136 (p=0.736)	0.13 vs 0.182 (p=0.871)
Hearing loss	0 vs 0	0.027 vs 0.027 (p=1.000)	0.09 vs 0.091 (p=1.000)	0 vs 0.027 (p=0.719)
Body temperature (score)	0.055 vs 0.027 (p=0.835)	0.491 vs 0.6 (p=0.835)	0.13 vs 0.082 (p=0.835)	0.17 vs 0.064 (p=0.719)
Body temperature (C)	29.73 vs 29.8182 (p=0. 835)	31.106 vs 30.948 (p=0.838)	29.192 vs 29.328 (p=0.835)	28.95 vs 29.39 (p=0.650)
Distended abdomen	0 vs 0	0 vs 0	0 vs 0	0 vs 0
Body condit on score	0 vs 0	0.073 vs 0.109 (p=0.850)	0.24 vs 0.155 (p=0.677)	0.24 vs 0.2636 (p=1.000)
Tumours	0.0909 vs 0.0682 (p=0.946)	0.2 vs 0.282 (p=0.835)	0.16 vs 0.055 (p=0.677)	0.16 vs 0.173 (p=1.000)
Prolapse (Penile)	0 vs 0	0 vs 0	0 vs 0	0 vs 0
Prolapse (Rectal)	0 vs 0	0 vs 0.027 (p=0.719)	0 vs 0	0 vs 0.027 (p=0.719)
Diarrhoea	0.027 vs 0.027 (p=1.000)	0.027 vs 0 (p=0.719)	0.03 vs 0 (p=0.720)	0 vs 0
Malocclusions	0.055 vs 0 (p=0.650)	0.027 vs 0.155 (p=0.652)	0.03 vs 0.218 (p=0.650)	0.07 vs 0.236 (p=0.718)
Forelimb grip strength (score)	0.182 vs 0.064 (p=0.719)	0.191 vs 0.164 (p=0.871)	0.15 vs 0.109 (p=0.835)	0.21 vs 0.082 (p=0.650)

Supplementary table 6B.

Assessment		Time point	CRT v PFB (p)
Grip Strength	**Forelimb grip strength (g)**	G1	101.24 vs 112.342 (p=0.719)
		G2	90.164 vs 91.73 (p=1.000)
		G3	78.217 vs 75.03 (p=0.929)
		G4	64.623 vs 74.776 (p=0.649)
Rotarod	**Rotat on (rpm)**	R1	9.15 vs 10.431 (p=0.834)
		R2	8.968 vs 11.263 (p=0.649)
	**Time (s)**	R1	42.897 vs 53.106 (p=0.719)
		R2	41.458 vs 60.152 (p=0.649)
	**Distance (m)**	R1	0.544 vs 0.675 (p=0.719)
		R2	0.501 vs 0.807 (p=0.649)

Supplementary table 6C.

Assessment		Time point	CRT v PFB (p)
Y-maze	**Spontaneous alternation**	Y1	0.643 vs 0.686 (p=0.721)
		Y2	0.63 vs 0.674 (p=0.736)
Barnes maze	**Primary latency_day1**	BM	59.703 vs 58.293 (p=1.000)
	**Total latency_day1**	BM	106.641 vs 116.433 (p=0.835)
	**Primary errors_day1**	BM	7.81 vs 9.2 (p=0.835
	**Total errors_day1**	BM	13.66 vs 22.11 (p=0.649)
	**Primary latency_day2**	BM	43.147 vs 45.492 (p=1.000)
	**Total latency_day2**	BM	73.044 vs 96.304 (p=0.665)
	**Primary errors_day2**	BM	7.438 vs 8.75 (p=0.834)
	**Total errors_day2**	BM	9.281 vs 18.796 (p=0.626)
	**Primary latency_day3**	BM	20.131 vs 20.257 (p=1.000)
	**Total latency_day3**	BM	31.575 vs 34.566 (p=0.871)
	**Primary errors_day3**	BM	4.719 vs 5.75 (p=0.835)
	**Total errors_day3**	BM	5.219 vs 9.5 (p=0.649)
	**Primary latency_day4**	BM	13.515 vs 17.475 (p=0.736)
	**Total latency_day4**	BM	21.541 vs 28.048 (p=0.692)
	**Primary errors_day4**	BM	5.219 vs 9.5 (p=0.650)
	**Total errors_day4**	BM	**3.375 vs 9.864 (p=0.011)**
	**Primary latency_day5**	BM	13.488 vs 11.655 (p=0.871)
	**Primary errors_day5**	BM	4.625 vs 3.727 (p=0.871)
	**Total errors_day5**	BM	28.625 vs 44.182 (p=0.649)
	**Primary latency_day12**	BM	30.95 vs 28.2 (p=1.000)
	**Primary errors_day12**	BM	6.625 vs 6.273 (p=1.000)
	**Total errors_day12**	BM	28.625 vs 38.364 (p=0.719)
